# Internet of things security evaluation mechanism based on meta attribute fluctuation

**DOI:** 10.1371/journal.pone.0282630

**Published:** 2023-07-14

**Authors:** Zhe Liu, Yinghao Yuan, Bo Zhao, Yixuan Wang

**Affiliations:** School of Cyber Science and Engineering, Wuhan University, Wuhan, 430072, China; Asia University, TAIWAN

## Abstract

In the field of Internet of Things (IoT), terminal security has always been an extremely important independent research topic. In the terminal security research, in addition to the security enhancement of terminal entities, the security status evaluation of terminal security has also become an independent subset of the security research in the IoT field. However, it should also be noted that the security attributes of IoT terminals can include many aspects, so judging the security of IoT terminals based on the overall security form is not enough for the security of terminal entities. This paper introduces the concept of volatility from the overall situation assessment to the meta attributes that constitute the overall security situation, and preliminarily realizes the construction of a concise model based on historical data to judge the meta attributes that may affect the overall security in the future. At the same time, a concise verification system is built based on the application scenario of the power IoT terminals currently under research to preliminarily realize trend prediction, further expand the trust evaluation of IoT terminals, and clarify the direction of further research.

## 1. Instruction

### 1.1 Research background

The concept of the IoT (IoT) was put forward in the 1990s and has been widely used [1,2]. In the field of power IoT, the network and information security of the power IoT directly relates to the production and operation security of the energy Internet, which is an important content to be considered for the network and information security of power grid companies [3]. By the end of 2018, the State Grid had access to a total of 540 million terminals [4,5], which has basically achieved the comprehensive collection of power grid operation control information and user electricity metering information. Considering the large-scale nature, multi-source heterogeneity, dynamic connectivity and high-speed mobility of the IoT, the IoT also has more security risks [6,7]. In the face of massive heterogeneous, multi-level cascaded IoT terminal equipment, in addition to ensuring its stable operation in the power IoT, it is also of great research significance to grasp the security situation of the terminal [8,9].

In the preliminary research field of power IoT security, KIMANI K and SHRESTHA M et al. [9,10] studied the characteristics of power IoT and the security threats and attack modes faced by all levels, proposed corresponding security protection measures, and proposed the construction of power IoT security protection framework. Wang et al. [11] proposed a security authentication method for smart grid terminals. This method layers the structure of smart grid terminal authentication system, improves the simplicity and scalability of system deployment, and realizes flexible communication mechanism, interaction mechanism between systems and integrity of terminal verification. Lu et al [12] comprehensively considered the physical layer, network layer and protocol layer anomaly characteristics of the terminal equipment, establish a portrait of the terminal equipment, depict the network access status of the terminal equipment, and combine specific attack scenarios to accurately identify the phishing and malicious terminal equipment, and achieve the security monitoring target of the heterogeneous full-service ubiquitous power IoT terminal.

However, some scholars have put forward different opinions on the security research of traditional methods in the IoT [13,14], and believe that traditional methods are difficult to solve the problem of internal attacks. F. A. Alaba et al. [13] summarized the security threats of the IoT and believed that the trust model can be introduced into the security assessment of the Internet of Things, but the current trust model of the Internet of Things has not yet formed the relevant work of establishing the trust mechanism. A. Ouaddah et al. [14] believe that, if the trust concept is used in the IoT field, the decentralized method supports trust better than the centralized method, because policies can be defined at the edge of the network, and no central entity needs to be introduced.

On the premise that traditional attacks are limited, the trust model is introduced into the IoT to better solve internal attacks. By establishing a trust model based on trust management and establishing trust attributes, you can use the internal interactive data of the IoT to evaluate the credibility of the terminal entity, thus judging the trust status of the terminal entity, and evaluating and selecting the security of the IoT terminal entity. In the past few years, scholars at home and abroad have proposed many trust models. By using fuzzy logic, graph theory, collaborative filtering, clustering analysis and other algorithms, direct trust and indirect trust are considered. Tang et al. [15] proposed a trusted cloud service selection framework combining objective trust assessment (QoS monitoring) and subjective trust assessment (feedback rating). This framework proposes a comprehensive trust evaluation method and applies it to the IoT. P. Varalakshmi et al. [16] aimed to select reliable service providers by evaluating reliability based on feedback from different sources, including customer feedback, global consulting feedback and third-party feedback.

Jian Wu [17] et al. proposed a trust based framework based on attitude trust model, harmony and consensus, which is used to establish a consensus recommendation mechanism for group decision-making with interval intuitionistic fuzzy information. A sensitivity analysis with attitude parameters is proposed to verify the rationality of the proposed attitude trust recommendation mechanism. This method helps inconsistent experts to achieve balance in the consensus field by selecting appropriate attitude parameters, thus forming a balance mechanism in the field of terminal security recommendation. Abderrahim O B et al. [18] proposed a centralized context based trust management system for the IoT. This system not only uses historical information to evaluate trust, but also receives feedback from the visited objects for evaluation. Yu Ning et al. [19] proposed an access control model suitable for cloud computing multi domain environment. This model can not only reduce the amount of access requests from high-risk users, but also meet the demand for dynamic authorization for users. However, the above two models are still limited by the single point of failure of the centralized evaluation model.

### 1.2 Research meaning

Based on the previous simple analysis of the current situation at home and abroad, it can be determined that the trust concept can effectively reduce the hardware requirements in the physical field of the IoT terminal, and can also reduce other hardware costs caused by the implementation of trusted computing. The trust score is a performance indicator based on the functional attributes related to the collaboration context. At the same time, it uses mathematical change ideas and frequency concepts to analyze the trust value, and further analyzes the possible security status of the IoT entities through trust. This paper expands the trust evaluation in the field of power IoT, and further evaluates the terminal entities based on the security elements that constitute the trust evaluation, so that the security evaluation of IoT terminals can adapt to a wider platform, and can also more objectively build the security system of IoT terminals based on the trust concept.

In this scheme, the calculation relationship between the trust element attributes and the overall trust degree is built by referring to previous experience on the basis of maintaining the overall trust degree. The main work and contributions are as follows:

First, Bayesian reasoning is introduced to calculate the trust element attribute value based on the data collected and transmitted by the IOT terminal entity, and the overall trust value is calculated based on the trust element attribute.

Secondly, based on the overall trust value and the trust element attribute, the trust change at each specific moment is calculated respectively, and the relationship between the overall trust value change and the trust element attribute change is established. Build the relationship between trust meta attributes and overall trust from multiple dimensions such as specific time and specific time period, and find trust meta attributes that are easy to lead to changes in overall trust.

Finally, the experiment is carried out using the general data acquisition simulation platform in the IoT field. The experimental results show that the improved scheme can effectively evaluate the credibility of IoT terminals, and even under several common malicious attacks (such as on-off attacks, transmission attacks and conspiracy attacks), it can also more effectively judge the state of trust element attributes.

The overall structure of this paper is as follows: The second part is the construction of the concept of attributes; The third part is the specific description of the improvement management plan; The fourth part is the trust analysis based on the above methods, and makes a horizontal comparison with other security assessment methods; The fifth part is the summary.

## 2. Evaluate the overall architecture design

In this section, we give a brief overview of the construction of the IoT terminal evaluation knowledge map based on trust meta attributes and trust fluctuations.

### 2.1 Evaluating host architecture concepts

**Fig 1:**
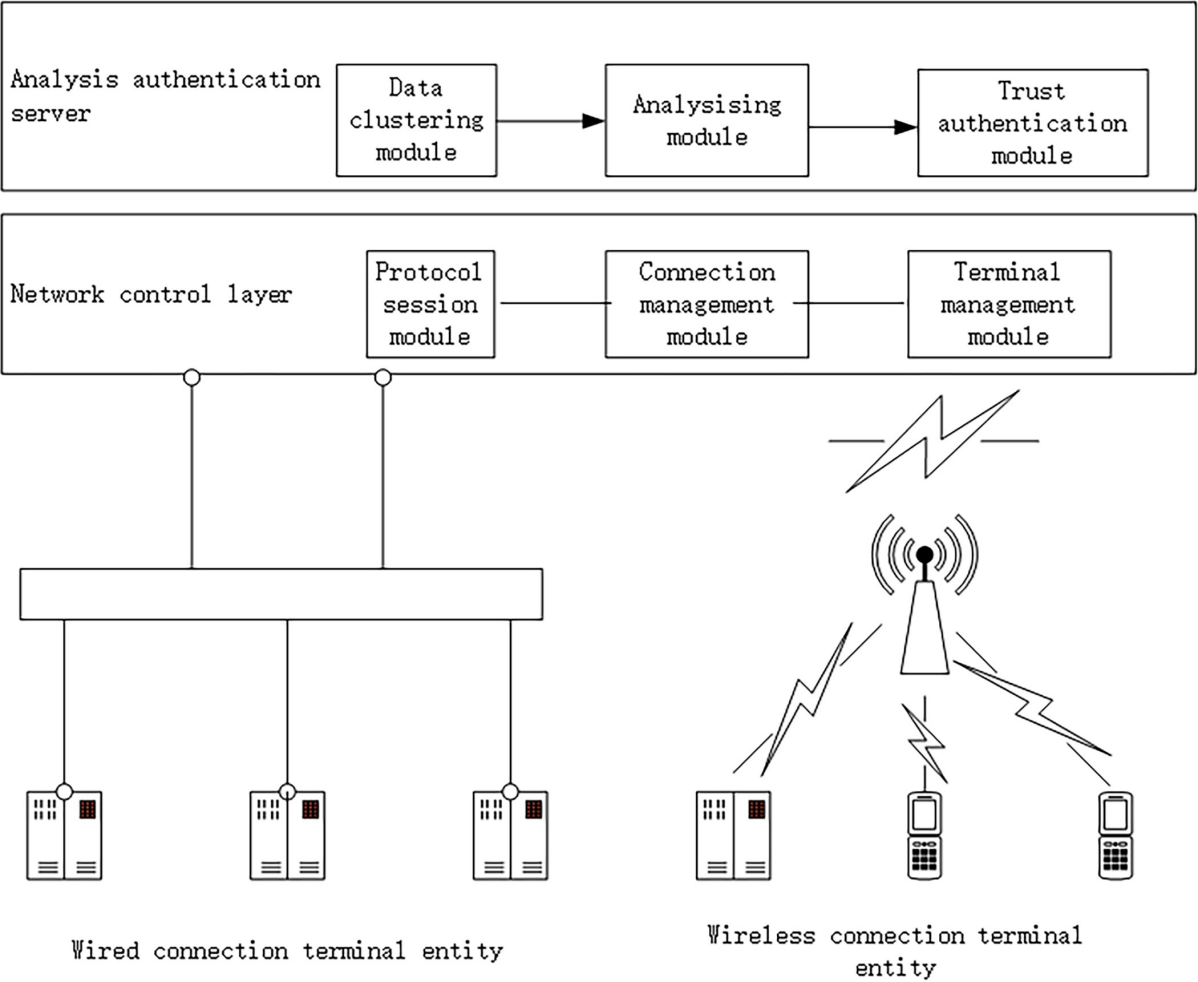
Network architecture.

Considering the most basic interaction requirements of IoT data, and referring to other architectures based on IoT trust analysis, this paper simplifies the IoT architecture into the following basic parts: power IoT terminal entity (PTE), data transmission network (DTN), and data trust analysis authentication server (TA). The specific structure is shown in Fig 1.

IoT terminal entity: In the scheme proposed in this paper, the data generated by the terminal entity is raw data that has not been processed. The above data is transmitted to the authentication server through the subsequent network for analysis and authentication. Considering that most of the terminal entities of the IoT have undergone preliminary security authentication in actual use, the above terminal entities are all in the default trusted state.

Data transmission network: through wired or wireless connection, the data generated by the terminal entity of the IoT can be completely transmitted to the authentication server. This network is called data transmission network. Considering that the IoT needs to transmit massive data and refer to other trust analysis hardware reservation, the traffic bandwidth reservation of the data transmission network is relatively large. At the same time, the data transmission network also needs to be responsible for taking anti transmission measures and other operations for the analyzed untrusted nodes.

Data analysis authentication server: this server plays an important role in verifying the trust status of terminal entities. In the design of this paper, the data analysis authentication server needs to receive the data uploaded by the terminal entity through the data transmission network, and use the data analysis algorithm to quantify the data into the attribute value of the trust element, and finally form the overall trust value of the terminal, and analyze the change of the trust value on the basis of the overall trust value and the attribute value of the trust element. In order to ensure the realization of the above functions, TA needs to have sufficient storage and computing resources. Therefore, this part is cloud platform by default.

### 2.2 The concept of meta attribute fluctuation

According to the overview of the overall scheme in 2.1, this section defines the relevant terms involved in the fluctuation of the construction meta attribute.

Trust level: trust level is based on the calculation conducted by various indicators of data interaction after the terminal entity completes the authentication, this paper uses trust to represent the user trust level, and the trust interval is set as [0,1].

Trust meta property: entity trust value t[C] represents the trust degree of an entity in the overall environment of the IoT under the IoT environment.

Default trust value: the trust status through preliminary security verification, the default trust value is set as 0.5 in this paper with reference to the more widely used normal distribution pattern in trust analysis in other fields.

Trust fluctuation: on the basis of trust fluctuation meta attribute, a mathematical expression between trust meta attribute and trust value is constructed. On the basis of the constructed mathematical expression, the idea of change rate is introduced to analyze the changing situation of trust meta properties at a time when trust values fluctuate greatly. If a change in the trust situation of a trust meta attribute induces a fluctuation in the trust value, then a certain trust meta attribute is considered sensitive to the trust situation of the terminal entity. If a small change in a trust meta attribute induces a large change in trust, the trust meta attribute has high sensitivity.

Trust fluctuation analysis model: on the basis of the constructed mathematical expression, the idea of change rate is introduced to analyze the changing situation of trust meta properties at a time when trust values experience substantial fluctuation; The results obtained from the analysis are divided into determining the point fluctuation of a certain trust meta attribute at a certain moment, depending on the moment, and the face fluctuation of the trend change of a certain trust meta attribute in a given time interval.

Fluctuation frequency: considering that the point fluctuation has a certain chance and the change of trust at part time may be the result of multiple meta attributes working together, so the concept of sensitive frequency is introduced in this paper to record the changing elements under the premise that trust changes substantially. In a given time frame, the number of changes in the trust meta properties that arise under the premise of a large change in trust, referred to as the trust fluctuation frequency. The more sensitive the trust meta property, known as trust fluctuation that is highest in a given time frame.

## 3. Construction of terminal security knowledge map based on trust meta attribute

This section is an overview of our proposed scheme, introduces how to calculate the trust meta attribute value and the overall trust value, and analyzes the sensitivity of the trust meta attribute and the overall trust.

Before introducing the definition of calculation in this chapter, we will first explain the symbols and definitions used in it, as shown in Table 1.

**Table 1:** Symbol definition table used in this paper.

Symbol	Definition
i	The I th node entity of the power IoT.
n	Number of messages received by the grid server.
k	Number of real messages received by the grid server.
*p*_*i*_	Node *v*_*i*_ The probability of sending a real message to the server, 0≤*p*_*i*_≤1
β	β distribution.
Γ	Gamma function.
α,β	The parameters of the gamma function, and α,β>0
*Td*	Total data.
*Ed*	Amount of leaked data.
*Fd*	Data volume of illegal operation.
*w*_*i*_	Transfer matrix value.
*s*_*i*_	The trust meta attribute set that constitutes the overall trust value.
*W* ^*T*^	The vector set composed of trust meta attributes.
*s*	The set of untrusted states.
*α*_2_	The damping coefficient.

### 3.1 Calculation of overall trust value

We assume that the overall trust value of the terminal entity of the IoT is composed of many trust meta attributes, and the trust meta attributes are selected according to the trust criteria, and there will also be continuous trust meta attributes and discrete trust meta attributes.

For discrete trust meta attributes, there are only two levels: trustable and untrustworthy. These two levels are represented by 1 and 0 respectively in trust evaluation. Considering that each evaluation of discrete trust element attributes is an independent process, the evaluation results follow binomial distribution. Suppose the server is from node *v*_*i*_ received n messages, of which K items are true. According to this standard, the likelihood function is described as follows:

$$f(k|v_{i})=C_{n}^{k}p_{i}^{k}{\left(1-p_{i}\right)}^{n-k}$$ (1)

*p*_*i*_ stands for node *v*_*i*_ is the frequency of sending authentic messages to the server, and the trust value of the discrete trust element attribute is related to this factor. However, considering that the direct introduction of 0 and 1 discrete trust element attributes into trust analysis can easily lead to sudden change of trust state, we change the evaluation system of discrete trust element attributes to a posterior distribution value with continuous attributes. Since the conjugate prior distribution of the binomial distribution obeys the beta distribution, we assume that the probability distribution of *p*_*i*_ is beta (α,β), And the prior distribution formula is given as follows:

$$f(p_{i};\alpha ,\beta )=\frac{\Gamma (\alpha +\beta )}{\Gamma (\alpha )\Gamma (\beta )}p_{i}^{\alpha -1}{\left(1-p_{i}\right)}^{\beta -1}$$ (2)

Γ Is gamma function, parameter α, β> 0 and 0≤*p*_*i*_≤1。 It should be noted that there is no interaction between the IoT terminal node and the trust server, and there is no prior knowledge. Therefore, it is assumed that Pi follows a uniform distribution, that is α = 1 and β = 1 special β Distribution.

According to Bayesian inference, the posterior distribution is given by the following formula:

$$f(p_{i}|k)\propto f(k|p_{i})f(p_{i})$$ (3)

By synthesizing the three Formulas (1–3), the posterior distribution formula obtained in this paper is shown in (4). Probability *p*_*i*_, and the expected value of *p*_*i*_ can be regarded as the terminal serve’’s response to the IoT entity v_ Trust meta attribute value m of *m*_*i*_。

$$f(p_{i}|k)=\frac{\Gamma (n+\alpha +\beta )}{\Gamma (k+\alpha )\Gamma (n-k+\beta )}p_{i}^{k+\alpha -1}{\left(1-p_{i}\right)}^{n-k+\beta -1}$$ (4)

According to the current analysis needs of the IoT and the previous selection of trust element attributes, this paper selects the discrete trust element attribute calculation method in the following element fields [18,19].

Reliability of communication link (K): the security of the link between the requester and the resource owner.

Normative behavior (B): whether the resource can be accessed according to the predetermined resource access mode.

For the continuous trust meta attribute, its trust value is a continuous value within the interval of [0,1], and the trust meta attribute value adopts a positive evaluation system. 0 means completely untrusted, and 1 means completely trusted. The initial states of continuous trust meta attributes are all equal, but they fluctuate with the change of interaction time.

According to the current analysis needs of the IoT and the selection of trust element attributes by predecessors, this paper selects the continuous trust element attribute calculation method in the following element fields.

Confidentiality (J): whether the confidentiality information obtained from the access can be guaranteed not to be disclosed.

Normalization of permission propagation (G): whether to transfer access permission only to trusted users.

On the basis of the trust vector, the trust vector is quantified into the trust value, which is the trust score of the trust meta attribute at a certain time. The calculation method of confidentiality is shown in Formula (5).


$$J_{i}=\left\{ \begin{array}{c} \frac{Td-Ed}{Td}\;If\;the\;data\;can\;be\;transmitted\;confidentiality\\ 0\;else \end{array}\right.$$ (5)

Formula (5) represents the definition of confidentiality. In the actual application environment of this paper, we use the proportion of data leakage to measure confidentiality. Where TD represents the total amount of data, and ED refers to the amount of data leaked.

Formula (6) is the definition of the trust meta attribute of authority propagation normalization. According to the terminal authority application under the IoT environment, the ratio of compliance authority application operations to total operations is taken as the definition of authority propagation normalization.


$$G_{i}=\left\{ \begin{array}{c} \frac{Td-Fd}{Td}\;When\;permissions\;can\;be\;executed\;as\;required\\ 0\;else \end{array}\right.$$ (6)

The overall trust value is an organic combination of multiple trust meta attribute values. Since the overall trust value is obtained by clustering quantification of trust meta attributes, the overall trust value does not simply decrease with time fluctuations, but becomes stable or fluctuates with the increase of data volume. The initial definition of trust in this paper is as follows:

$$T[i]\equiv (J_{i}{,\;G}_{i},\;K_{i},\;B_{i})$$ (7)

When building the relationship between the overall trust value and the trust meta attribute value, we use the transfer matrix to represent the transfer of the trust meta attribute in given time t. Transfer matrix value *w*_*i*_ can be obtained by the following formula:

$$w_{i}=\left\{ \begin{array}{c} m_{i}\;If\;m_{i}\neq 0\\ 0\;else \end{array}\right.$$ (8)

*m*_*i*_ represents the element value of the previous trust meta attribute, and *m*_*i*_ is selected from the four trust meta attributes (*J*_*i*_, *G*_*i*_, *K*_*i*_, *B*_*i*_). Since data transmission starts from the default trust value, as long as the total trust value is not zero, we believe that there is a trust relationship between the trust population and the trust meta attribute. *w*_*i*_ is shown in Formula (9):

$$w_{i}=\frac{w_{i}}{\sum_{i\in s_{i}}w_{i}}$$ (9)

*s*_*i*_ is the trust meta attribute set that constitutes the overall trust value. However, considering that once the attribute of discrete trust element is zero, there may be no definition according to the above definition, so this paper uses the definition of the prior posterior distribution to calculate the trust value based on their trust history. At this time, the trust value is determined by the old discrete trust element attribute value.

Based on the above matrix definition, the concept of the overall trust value in this paper is that the overall trust state starts from the default trust. With the passage of time and the increase of the data volume, the trust state will fluctuate. The definition formula is as follows:

$$T[i]=\alpha_{2}∙W^{T}+(1-\alpha_{2})∙s$$ (10)

In the above formula, *W*^*T*^ represents the vector set composed of trust meta attributes, and s represents the set of untrusted states. *α*_2_ is the damping coefficient, where 0 <*α*_2_<1。 The damping coefficient increases with the increase of measurement time, which basically conforms to the application status of IOT terminals.

### 3.2 Analysis of trust fluctuation

In the field of the IoT, because the overall trust value has some chance, in order to objectively measure the trust state from the perspective of time change, we refer to Birnbaum based measurement methods in social networks (Zhao et al. [20]; Akers [21]) and define trust fluctuation as the partial derivative of the overall trust value or trust meta attribute based on time t during data interaction. According to the different objects of fluctuation measurement, we divide the fluctuation into the overall trust value fluctuation and the trust meta attribute value fluctuation. If the overall trust value fluctuates, at least one trust meta attribute will fluctuate, and it is positively related to the overall trust value. Considering that the overall trust value T[i] is composed of four trust meta attributes, according to Formula (7), the trust value T[i] is composed of (*J*_*i*_, *G*_*i*_, *K*_*i*_, *B*_*i*_). After the trust meta attribute is introduced into the overall trust value, the fluctuation definition of the overall trust value is as follows:

$$ST[i]=\frac{dT[J_{i}{,\;G}_{i},\;K_{i},\;B_{i}]}{di}$$ (11)

*ST*[*i*] represents the trust fluctuation of the overall trust value t at time I. if the fluctuation of the overall trust value is relatively weak, it can be considered that the trust state of the terminal entity at a specific time is relatively stable.

Because the trust value is processed continuously in this paper, the default four types of trust meta attributes are all continuous trust after mathematical deduction, and the corresponding is that there are k different trust states between the overall trust value and the trust meta attribute value.

In the fluctuation analysis, the overall trust value fluctuation is obtained by the fluctuation of each trust element attribute. According to this assumption, *ST*[*i*] can be expressed as Formula (12).


$$\left\{ \begin{array}{c} ST[i]=\frac{\partial J_{i}}{\partial i}di+\frac{\partial G_{i}}{\partial i}di+\frac{\partial K_{i}}{\partial i}di+\frac{\partial B_{i}}{\partial i}di\\ dJ(i)+dG(i)+dK(i)+dB(i)=0 \end{array}\right.$$ (12)

According to Formula (12), at this time, the trust fluctuation at time I can be calculated, but for the measurement state, the fluctuation at a certain time cannot fully represent the overall fluctuation in the time period. In order to measure the impact of trust fluctuation from the spatial dimension, we introduce the concept of frequency, and introduce the graph theory of trust relationship into the analysis of overall trust fluctuation.

On the basis of Formula (12), this paper designs an algorithm based on the change angle to realize the fluctuation analysis under the condition of sudden change of trust state.

## 4. Experiment and analysis

In this section, we use the settings of evaluation experiments in the common IOT data acquisition simulation platform, and introduce the evaluation results of our proposed model. At the same time, some attacks are simulated, and the proposed scheme is compared with the existing trust analysis schemes to verify the anti attack and objectivity of the trust analysis model based on trust fluctuation.

### 4.1 General introduction of the experiment

**Fig 2:**
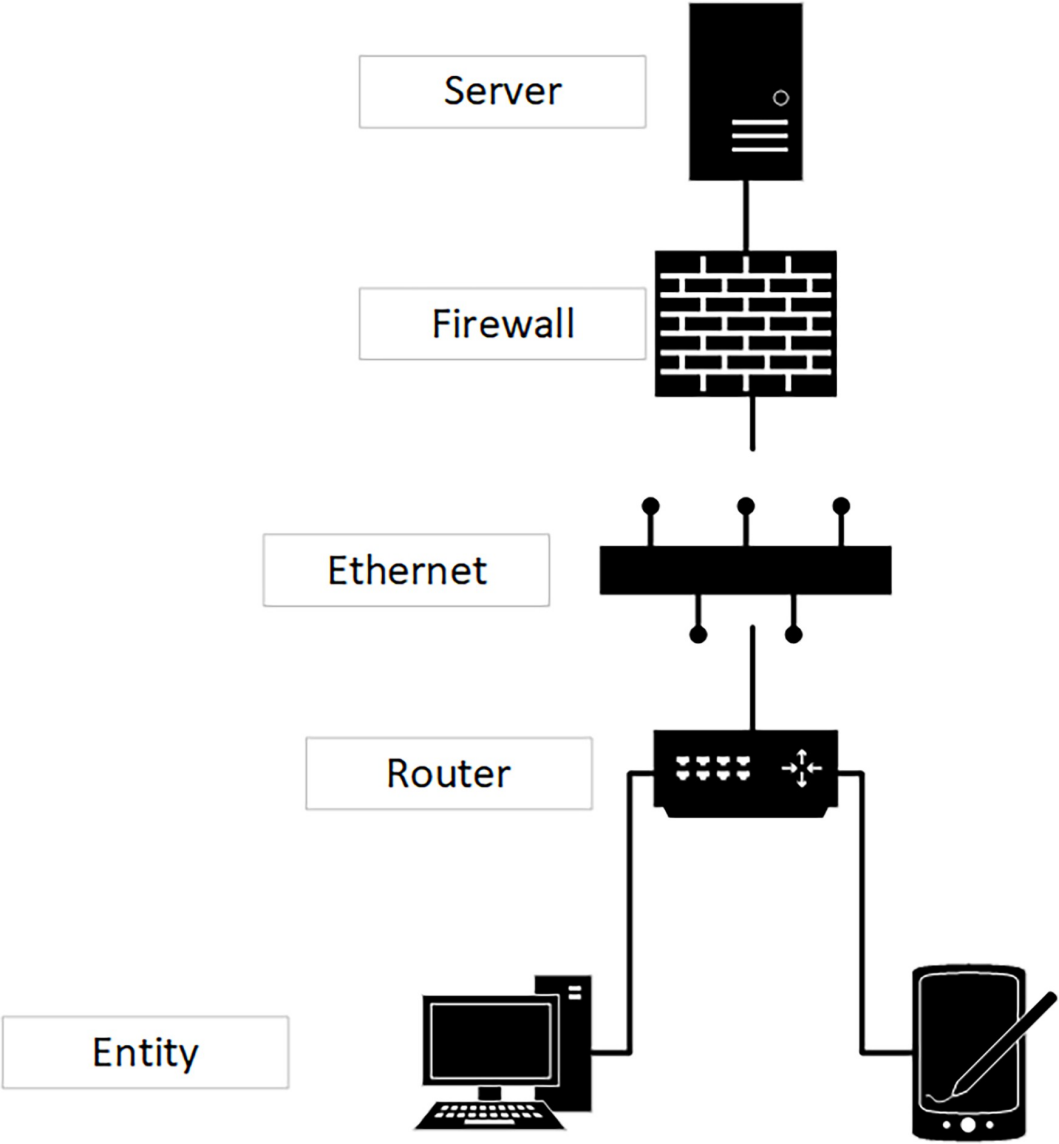
Topology of simulation evaluation.

The edge IoT dataset used in the experiment contains the data information related to the IoT, and the security situation attribute set required by the prediction method is extracted from the information of the dataset, and is used as a knowledge map for security situation judgment. First, the network topology of each part of the network is shown in Fig 2. The topology can be obtained with the help of automatic topology discovery tools, such as object SNMP.

Although the scheme proposed in this paper can be used in a variety of application scenarios, our focus is on real-time measurement of the behavior logic of IOT terminal entities in the data collection stage. To be more specific, we set four basic trust evaluation elements according to the data collection behavior of the IoT, and integrated the trust evaluation elements to form a trust degree. If the overall trust value is lower than the default trust value of 0.5, we think it is not trustworthy; If the trust fluctuates greatly, we also think that the entity stability is poor. In the simulation, referring to the idea of normal distribution, we set 0.5 as the default trust value, and believe that the trust state higher than 0.75 is higher.

At the same time, we refer to the relevant characteristics of dynamic trust mentioned by predecessors [22], including no change, stable positive, stable negative, blind positive, blind negative, slow positive but fast negative, and slow negative but fast positive. We also take the relevant characteristics of dynamic trust as the basis for sensitivity analysis.

In order to verify the improvement and security of the scheme, this part compares the proposed improvement models under various trust States, and the evaluation indicators proposed above measure the above models.

### 4.2 Analysis and prediction based on meta attribute fluctuation

In this part, we mainly verify the trust fluctuation when the overall trust value is in a stable state, and make a simple assessment of the robustness of the overall trust evaluation. Specifically, we start with the overall fluctuation and further analyze the changes in the attributes of each trust element of the terminal entity on the premise of the overall large fluctuation.

Step 1: Select stable trust fluctuation.

In the actual evaluation process, the overall trust fluctuation can be divided into two forms: relative stability and relative jump. For relatively stable terminal entities, the security fluctuation of meta attribute has no actual impact on the whole. Therefore, we will not do too much analysis on the entity interval with small fluctuation and no cross domain fluctuation in this paper.

Step 2: Select large trust fluctuations.

In this process, we calculate the trust fluctuation of the terminal entity and count the trust time periods with large fluctuations, as shown in Table 2. Table 2 shows the trust value, and shows the trust fluctuation range in which the overall trust value is located.

**Table 2:** Overall trust fluctuation (each minute).

Time	1	2	3	4	5	6	7	8	9	10
Trust(T)	0.50	0.54	0.58	0.51	0.60	0.78	0.64	0.79	0.67	0.58
Trust Change (T’)	-	0.04	0.04	-0.07	0.09	0.18	-0.14	0.15	-0.12	-0.09
Time	11	12	13	14	15	16	17	18	19	20
Trust(T)	0.69	0.66	0.53	0.65	0.61	0.53	0.58	0.64	0.56	0.67
Trust Change (T’)	0.11	-0.03	-0.13	0.12	-0.04	-0.08	0.05	0.06	-0.08	0.11

Table 2 shows the trust fluctuation of the power Internet of Things terminal entity. According to the trust values shown in the table, the trust values of the entity are in the default trust state. In terms of traditional trust analysis methods, this basically means that the evaluation has been completed.

We write the trust fluctuation at each time in Table 2, and preliminarily concluded that the change trend of the entity was relatively obvious. Although the overall trend from the beginning of measurement to the final deadline is rising, the performance is not consistent at each time point. For example, the fluctuation pattern of the first three points is a steady rise, and the overall fluctuation is not stable at 7–14 points. Even if they are in the discrete interval of default trust, the large fluctuation of trust value is not a very stable security form. Although the overall trend is increasing in a certain period of time, its increasing form and trend are not stable, so such entities need further evaluation and analysis.

Step 3: Calculate the fluctuation value of each meta attribute under the condition of overall large fluctuation.

According to the definition mentioned in the previous work, the trust meta attribute has a positive effect on the overall trust, and at least one meta attribute will have an impact on the overall trust under the condition of large overall fluctuations. According to this principle, Table 2 shows the value of trust value of each meta attribute under the premise of large fluctuation, and Table 4 calculates the changes of the attributes of each trust element under the condition of large fluctuations.

In Table 3, it is clear that during the above time period, the overall trust value of the terminal entity and each attribute value constituting the overall trust are trusted by default, and some of the attributes can reach a highly trusted form. However, it is not possible to fully judge the impact of each element on the fluctuation of the terminal entity, so we have further classified it into Table 4.

**Table 3:** The value of each meta attribute trust value under the premise of large fluctuations. (Designated minute).

Time	6	7	8	9	11	13	14	20
Trust(T)	0.78	0.64	0.79	0.67	0.69	0.53	0.65	0.67
TrusI)	0.65	0.62	0.68	0.61	0.64	0.56	0.70	0.70
Trust(G)	0.66	0.63	0.67	0.65	0.71	0.61	0.69	0.69
Trust(K)	0.81	0.75	0.82	0.75	0.71	0.52	0.63	0.66
Trust(B)	0.89	0.66	0.83	0.64	0.63	0.50	0.65	0.71

**Table 4:** Fluctuation of each meta attribute under the premise of large fluctuation. (Designated minute).

Time	6	7	8	9	11	13	14	20
Trust Change (T’)	0.18	-0.14	0.15	-0.12	0.11	-0.13	0.12	0.11
TruI (C)	0.03	-0.03	0.06	-0.07	0.01	-0.04	0.14	0.00
Trust^,^ (G)	0.02	-0.03	0.04	-0.02	0.02	-0.05	0.08	0.06
Trust^,^ (K)	0.16	-0.06	0.07	-0.07	0.04	-0.07	0.11	0.08
Trust^,^ (B)	0.19	-0.23	0.17	-0.19	0.13	-0.13	0.65	0.14

In Table 4, if we take the most traditional trust state at a certain time as the first benchmark for trust evaluation, then the state of the entity before the trust mutation is consistent with the current basic trust state. Taking the 6 and 7 minute moments as examples, normative behavior (B) brings the largest fluctuation when the entity rises or falls significantly. Therefore, at this time, we can think that the normative behavior has the greatest impact on the trust security of the entity at the above time.

Under the premise of using the same evaluation index system, this part mainly analyzes the ratio of the time spent in calculating the trust value to the trust value evaluation system. The figure shows that our method is superior to other methods in the following aspects: the time complexity based on fluctuation is relatively minimum, and because this paper does not use the weighted algorithm according to the grid survey, the relative time complexity of the method used in this paper is lower than that of the weighted method.

In addition, the introduction of the weighting algorithm increases the time complexity of the classification system, and because of the existence of the weight and it is difficult to change with the state of the entity, this may also cause uncertainty to the result of the trust value, and it is difficult to determine the accuracy and real-time, which ensures the evaluation of trusted entities.

## 5. Summary

In the application scenario of the IoT, because the terminal of the IoT has the characteristics of mass, multi-source heterogeneity, dynamic connection and high-speed mobility, and the traditional security encryption scheme can only defend against external attacks, the trust model is introduced to improve the internal security. And because the current trust model has certain limitations in terms of weight and real-time, this paper makes improvements to the existing trust management scheme of the IoT.

Firstly, the total trust and trust meta attribute are introduced to construct the calculation relationship between trust meta attribute and total trust.

Secondly, Bayesian inference is introduced to calculate the trust meta attribute value based on the data collected and transmitted by the terminal entities of the IoT, and the overall trust value is calculated based on the trust meta attribute. On the basis of the overall trust value and the trust meta attribute value, the trust change at each specific moment is calculated separately, and the relationship between the overall trust value change and the trust meta attribute change is built.

The relationship between th” tru’t meta attribute and the overall trust is built from multiple dimensions such as the specific moment and the specific time period, and the trust meta attribute that is easy to cause the overall trust change is found. Finally, the commonly used simulation model of the IoT is used for experimental verification. The results show that, through the above simulation experiments, the scheme proposed in this paper can find out the IoT terminals that may fluctuate from the perspective of change in addition to the trust element attribute and trust value, and has the feasibility of promotion in the field of IoT.

Although this paper builds an improved trust evaluation model from the perspective of volatility, and initially realizes the security trend evaluation by building a knowledge map, this is only the first step, and further exploration is needed in the fields of thresholds and standards. At the same time, due to a large number of interactions among entities in the IoT, interaction, a realistic factor, also needs to be further analyzed and studied from the perspective of fluctuations. Finally, it is also hoped that from the category of the IoT to more category systems of the IoT, this concept can be actually promoted and better applied.

## Supporting information

S1 File (DOCX)
