# Supplementary material for: Internet of things security evaluation mechanism based on meta attribute fluctuation
Source: PLoS One. 2023 Jul 14;18(7):e0282630. doi: 10.1371/journal.pone.0282630 (PMC10348552; doi:10.1371/journal.pone.0282630)
Supplement: S1 File — (DOCX) [file pone.0282630.s001.docx]

This part of the data is the original of our previous experiment, which is attached to the article in the form of text schedule in this revision. Here, the data used for analysis shall be separated into volumes as required, and the table notes appearing in the text shall be reserved for reference and analysis in the future. Among them, Trust（T）represents the obtained data, and Trust Change($T^{,}$) represents the change amplitude data after calculation and analysis.

At the same time, it should be added that the above data represent a stage of trust implementation evaluation. In a variety of entities, the trust value measured in real time will also change due to various factors, but the method used can still be used to evaluate the change status.

Table 2 Overall trust fluctuation (each minute)

| Time | 1 | 2 | 3 | 4 | 5 | 6 | 7 | 8 | 9 | 10 |
| --- | --- | --- | --- | --- | --- | --- | --- | --- | --- | --- |
| Trust(T) | 0.50 | 0.54 | 0.58 | 0.51 | 0.60 | 0.78 | 0.64 | 0.79 | 0.67 | 0.58 |
| Trust  Change  ($T^{,}$) | - | 0.04 | 0.04 | -0.07 | 0.09 | 0.18 | -0.14 | 0.15 | -0.12 | -0.09 |
| Time | 11 | 12 | 13 | 14 | 15 | 16 | 17 | 18 | 19 | 20 |
| Trust(T) | 0.69 | 0.66 | 0.53 | 0.65 | 0.61 | 0.53 | 0.58 | 0.64 | 0.56 | 0.67 |
| Trust  Change  ($T^{,}$) | 0.11 | -0.03 | -0.13 | 0.12 | -0.04 | -0.08 | 0.05 | 0.06 | -0.08 | 0.11 |

Table 3 The value of each meta attribute trust value under the premise of large fluctuations.(Designated minute)

| Time | 6 | 7 | 8 | 9 | 11 | 13 | 14 | 20 |
| --- | --- | --- | --- | --- | --- | --- | --- | --- |
| Trust(T) | 0.78 | 0.64 | 0.79 | 0.67 | 0.69 | 0.53 | 0.65 | 0.67 |
| Trust(C) | 0.65 | 0.62 | 0.68 | 0.61 | 0.64 | 0.56 | 0.70 | 0.70 |
| Trust(G) | 0.66 | 0.63 | 0.67 | 0.65 | 0.71 | 0.61 | 0.69 | 0.69 |
| Trust(K) | 0.81 | 0.75 | 0.82 | 0.75 | 0.71 | 0.52 | 0.63 | 0.66 |
| Trust(B) | 0.89 | 0.66 | 0.83 | 0.64 | 0.63 | 0.50 | 0.65 | 0.71 |

Table 4 Fluctuation of each meta attribute under the premise of large fluctuation

(Designated minute)

| Time | 6 | 7 | 8 | 9 | 11 | 13 | 14 | 20 |
| --- | --- | --- | --- | --- | --- | --- | --- | --- |
| Trust Change ($T^{,}$) | 0.18 | -0.14 | 0.15 | -0.12 | 0.11 | -0.13 | 0.12 | 0.11 |
| Trust^,^ (C) | 0.03 | -0.03 | 0.06 | -0.07 | 0.01 | -0.04 | 0.14 | 0.00 |
| Trust^,^ (G) | 0.02 | -0.03 | 0.04 | -0.02 | 0.02 | -0.05 | 0.08 | 0.06 |
| Trust^,^ (K) | 0.16 | -0.06 | 0.07 | -0.07 | 0.04 | -0.07 | 0.11 | 0.08 |
| Trust^,^ (B) | 0.19 | -0.23 | 0.17 | -0.19 | 0.13 | -0.13 | 0.65 | 0.14 |
